# Ostracism Predicting Suicidal Behavior and Risk of Relapse in Substance Use Disorders

**DOI:** 10.7759/cureus.61519

**Published:** 2024-06-02

**Authors:** Habiba Ali, Muddsar Hameed, Mahrukh Anwar Abbasi, Alishba Ali, Zamurd Abbas, Cijal Rahim Valiyakath, Gohar Ahmad Abbasi, Aliyah Usman Qureshi, Maryam Kainaat, Alishba Amer

**Affiliations:** 1 Department of Clinical Psychology, University of Karachi, Karachi, PAK; 2 Department of Clinical Psychology, Shifa Tameer e Millat University, Islamabad, PAK; 3 Department of Internal Medicine, Foundation University Medical College, Rawalpindi, PAK; 4 Department of Speech Pathology, Children's Hospital, Lahore, PAK; 5 Department of Medicine, International European University, Bishkek, KGZ; 6 Department of General Practice, Guangxi Medical University, Nanning, CHN; 7 Department of Medicine, Shifa Tameer e Millat University, Islamabad, PAK; 8 Department of Physiotherapy, Rawalpindi Medical University, Rawalpindi, PAK; 9 Department of Management, National University of Modern Languages, Islamabad, PAK; 10 Department of Nutrition, Lahore Medical and Dental College, Lahore, PAK

**Keywords:** social support, substance use disorders, relapse, suicidal behavior, ostracism

## Abstract

Introduction: The present study investigated the predictive relationship between ostracism and suicidal behaviors in individuals with substance use disorders. It also attempts to highlight the mediating role of the risk of relapse between ostracism and suicidal behavior.

Methods: The study was based on a cross-sectional survey design. The sample comprised 100 men aged between 30 to 45 years (M = 35.25, SD = 3.06) from Karachi. The purposive sampling technique was employed. The study employed demographic forms and three self-reporting measures: the Ostracism Experience Scale (OES-A), the Advance Warning of Relapse Questionnaire 3.0 (AWARE), and the Suicide Behaviors Questionnaire-Revised (SBQ-R).

Results: Ostracism significantly predicted relapse risk and suicidal behavior. Risk of relapse positively predicted both dimensions of ostracism (ignored: r = 0.33, p < 0.01; excluded: r = 0.43, p < 0.01) and suicidal behavior (r = 0.35, p < 0.01). Additionally, the risk of relapse strongly correlated with overall ostracism score (r = 0.43, p < 0.01). However, no significant mediating effect of ostracism on suicidal behavior was found. The effect was mediated through the risk of relapse (B indirect = 0.12, 95% CI = 0.04, 0.23). These findings suggest that ostracism increases the likelihood of recurrence, which in turn is associated with suicidal behavior. The mediation model explained 17% of the variation in suicidal behavior.

Conclusion: The findings propose the importance of addressing ostracism as a risk factor for suicidal behavior and relapse in substance use disorders. The results suggest that reducing the adverse effects of ostracism and improving social support for individuals can have a significant impact on their mental health.

## Introduction

Suicidal behavior is common among individuals with chronic conditions, and interventions aim to address this through preventative techniques. Suicide is a leading cause of premature death in young adults [1]. Suicidal thoughts are often caused by the ostracizing conduct that people with substance use disorders (SUD) experience, as evidenced by recent empirical research [2]. The interpersonal theory of suicide (IPTS) suggests that suicide is driven by a desire to die, thwarted belonging, and feeling like a burden, leading to suicidal thoughts [3]. Stable social connections are crucial for mental health, as social exclusion, such as ostracism, can affect self-perception and increase the likelihood of relapse. SUD, also known as drug addiction, is a significant national concern, causing substantial discomfort or disability.

Ostracism, a term originating from ancient Greek practices, refers to ignoring and excluding individuals or groups, often through refusal to communicate. This social exclusion is a common issue for individuals with SUDs, even after recovery, and is a significant factor in the risk of relapse. Factors contributing to ostracizing behavior include stigma and discrimination. It is considered one of the prominent factors responsible for the risk of relapse, and hence, interventions have been designed to address social exclusion for recovered patients [4].

Social psychology explores ostracism, a form of exclusion that reduces fundamental needs like belonging and self-esteem, impacting depression and loneliness. Ostracism, a form of discrimination, can lead to adverse effects such as psychological pain, physical health issues, and depression, compromising one's ability to recognize and control their social environment. This negatively impacts a person's sense of purpose, value, and self-worth, leading to a loss of social connections and jeopardizing their overall sense of meaning. Repeated experiences of ostracism and rejection can lead to increased sensitivity to rejection signals and a tendency to withdraw from social interactions, which, while reducing the risk of further rejection, can also result in loneliness and alienation. Loneliness is often a result of learned helplessness and alienation and is positively associated with depression [5].

The temporal need-threat model of ostracism shows a strong link between the risk of relapse and suicidal thoughts and actions, as well as the many signs of ostracism. The three-step theory (3ST) suggests that perceived burdensomeness, failed belongingness, and learned competence significantly influence suicidality levels, with high burdensomeness and low belonging predicting suicidal ideation [6].

Regarding drug use, it can occur at any time during a 12-month period and can involve both illegal and licensed medications. However, drug use does not necessarily equate to misuse, and some individuals may never develop dependence on drugs due to supportive social situations [7]. The DSM-5 acknowledges that drug addiction may be caused by low self-control, while SUD, as defined by the American Psychiatric Association, involves compulsive drug use without considering its negative consequences [8].

Despite the fact that addiction is considered a chronic illness, relapse is viewed as a natural part of the recovery process. Relapse refers to the recurrence of a disease that has gone into remission or recovery. Recovery may change social needs, causing individuals to seek support from those helping them recover [9]. As a chronic disease, addiction is subject to periods of relapse. Identifying these symptoms can help prevent relapses, as they often occur along the way. Researchers have identified risk factors for relapse in addiction, including destructive thoughts, compulsive behavior, neglect of coping skills, unhealthy environments, mood swings, depression or anxiety recurrence, and isolation from groups and activities, also known as 'ostracism' [10].

This study explores the link between ostracism, risk of relapse, and suicidal behavior in individuals with SUDs. Ostracism increases the risk of relapse and worsening mood problems, while social exclusion can evoke pain and threaten psychological needs [11].

The rationale of the study

The risk of relapse and suicidal thoughts in people with drug use disorders has been extensively studied. For instance, a recent study suggests that interactions with former substance users, adverse family reactions, inability to control urges, and work/social stress are the leading causes of relapse [12]. Similarly, social exclusion, which is one form of ostracism, is also related to the escalated rate of suicidal behavior. Others have observed that social isolation is positively related to relapse risk, and relapse prevention techniques may include family and friend assistance [13]. This evidence suggests that ostracism is a triggering factor for relapse in people with SUDs. Furthermore, it is also related to an increase in suicidal behaviors due to similar underlying cognitive processing. Literature has found a variety of risk factors for suicidal behavior, including adultery, depression, and social taunting [14].

The majority of individuals with SUDs in Pakistan are aged 25-39 years, with cannabis usage highest among 30-34 and heroin use highest among 35-39 year-olds [15]. These age groups are the most productive, and interventions to minimize dependency are needed. This research focuses on 30-45-year-olds, exploring the role of ostracism and the mediating role of relapse risk in this population.

Significance of the study

The study is essential as it will address the substance use problem in a population that has the potential to become the most productive workforce in the nation. Empirical literature shows that the variables of the study, including ostracism and risk of relapse, are correlated with the study outcome, i.e., suicidal behavior [16]. However, the predictive role of ostracism in suicidal behavior is not well investigated. Identifying the risk factors for suicidal thoughts in individuals with drug use disorders may reduce the number of suicides. Furthermore, the findings will help to develop interventions for effective management of the risk of relapse for individuals with SUDs.

The significance of this study lies in its ability to shed light on the potential impact of ostracism on individuals with SUDs, specifically regarding suicidal behavior and the risk of relapse. SUD is a major public health concern, and individuals with SUDs are already at a higher risk for suicidal behavior and relapse. This study hypothesizes that ostracism is a predictor of these outcomes in individuals with SUDs.

Moreover, SUDs are often accompanied by mental health problems, including depression, anxiety, and trauma. Ostracism can exacerbate these problems and contribute to a cycle of hopelessness, isolation, and thoughts of suicide. This study could provide important insight into the factors contributing to suicide and relapse in this population. This knowledge can inform the development of interventions and support systems that consider the negative impact of ostracism on mental health. Furthermore, the study could help destigmatize the condition and increase awareness of the complex and multifaceted nature of the disorder. By identifying ostracism as a risk factor, the study may help improve the quality of life for individuals with SUDs and ultimately reduce the incidence of suicide and relapse among this population.

This study aims to investigate the predictive relationship between ostracism and suicidal behaviors in individuals with SUDs. It also examines the mediating role of the risk of relapse in this relationship. Additionally, the study seeks to highlight the importance of addressing ostracism and enhancing social support to improve mental health outcomes for this population.

## Materials and methods

The research sample consisted of 100 participants with an age range from 30 to 45 years (M=35.25, SD=3.06) and individuals with SUDs. Participants with minimum matriculation level of education were included. All participants were from follow-up groups with polysubstance use and belonged to middle socioeconomic status from different treatment and rehabilitation centers situated in Karachi. After informing participants about the purpose of the study, informed consent was obtained before they voluntarily participated.

Following are the inclusion and exclusion criteria used to choose the sample. Adult men (aged 30-45) participated in the research. Follow-up clients of polysubstance use and belonging to middle socioeconomic status were included in the study. Only married males with SUDs were selected for the study. Only those patients who had received therapy for at least one month were considered eligible for inclusion. Individuals who had relapsed to use drugs at least twice were considered as relapse cases. Participants who could not understand the directions presented to them to complete the study questionnaire were excluded. Participants who were unable to complete the questionnaire were excluded. Participants who had any physical disability were excluded.

The research procedure utilized several tools: a consent form, a demographic form, and three specific instruments. The Ostracism Experience Scale (OES), as presented in Appendix A, an 11-item five-point Likert scale with an alpha reliability of 0.97, provided insights into social exclusion [17]. The Advance Warning of Relapse Questionnaire 3.0 (AWARE), as depicted in Appendix B, refined to include 28 items on a seven-point Likert scale, effectively predicted relapse (r = 0.42, p < 0.001) following outpatient alcohol treatment [18]. Meanwhile, the Suicide Behaviors Questionnaire-Revised (SBQ-R), as shown in Appendix C, a four-item scale with high internal consistency (α = 0.97), offered a concise yet robust self-report measure for past suicidal behaviors [19]. Collectively, these instruments facilitated comprehensive exploration within the research domain.

Procedure

The research was first approved by the Institute of Clinical Psychology, University of Karachi's departmental research committee (IRB-ICP-2029). After that, permission was sought from the concerned authority of treatment centers, and a permission letter was duly signed to grant permission. Data collection from the sample participants was done on an individual basis. The sample was approached through a purposive sampling technique during the research process. They were also informed about their right to withdraw from the research at any stage. They were further instructed to sign the consent form after reading it thoroughly. They were also assured that the information they provided would be kept confidential. The OES-A was presented first. The participants' average time to complete the first questionnaire was 5-10 minutes, whereas the average administration time was 10-15 minutes for completing the second and third questionnaires. The results from the scored questionnaires were analyzed using SPSS software v. 26.0 (IBM Corp., Armonk, NY).

## Results

This section describes the statistical analysis of the gathered data using v. 26 of the SPSS software. The findings are below.

Table 1 shows descriptive data of study variables and psychometric properties of instruments. Internal consistency is assessed with Cronbach’s alpha. The results indicate that all the scales have good internal consistency, with the alpha ranging from α = 0.74 to 0.85. Mean and standard deviation values show that the data is well distributed, which is also confirmed by skewness and kurtosis values.

**Table 1:** Descriptive statistics of study variables (N =100). Alpha= Cronbach's alpha, SD=standard deviation, skew=skewness, kurt=kurtosis

	No. of Items	Alpha	Mean	SD	Range		Skew	Kurt
					Potential	Actual		
Risk of relapse	26	0.76	156.67	9.13	26-182	131-177	-0.43	-0.25
Suicidal behavior	4	0.74	12.19	3.02	3-18	5-16	-0.27	-1.07
Ostracism-ignored	5	0.85	9.30	2.84	5-25	5-19	1.03	0.49
Ostracism-excluded	6	0.84	22.81	2.06	6-30	16-25	-1.22	1.70
Ostracism, total	11	0.81	31.95	3.77	11-55	23-40	0.64	0.48

Table 2 shows the Pearson correlation coefficients among the study variables. Age does not show a significant correlation with any other variable. Age of onset has weak correlations with risk of relapse (r = 0.08) and suicidal behavior (r = -0.18), but these are not statistically significant. The risk of relapse is positively correlated with suicidal behavior (r = 0.35, p < 0.01), ostracism-ignored (r = 0.33, p < 0.01), and overall ostracism score (r = 0.43, p < 0.01). Suicidal behavior shows significant positive correlations with both ostracism-ignored (r = 0.26, p < 0.05) and ostracism-excluded (r = 0.24, p < 0.05). The strongest correlation is observed between ostracism-ignored and ostracism-excluded (r = 0.79, p < 0.01), indicating a close relationship between these forms of ostracism. These findings emphasize the interconnectedness of relapse risk, suicidal behavior, and social ostracism.

**Table 2:** Pearson bivariate correlations among study variables (N =100). * p < 0.05, ** p < 0.01 was considered of statistical significance.

Serial No.	Variables	1	2	3	4	5	6
1	Age	-	-	-	-	-	-
2	Age of onset	0.08	-	-	-	-	-
3	Risk of relapse	0.11	-0.03	-	-	-	-
4	Suicidal behavior	0.17	-0.18	0.35^**^	-	-	-
5	Ostracism-ignored	-0.11	-0.04	0.33^**^	0.26^*^	-	-
6	Ostracism-excluded	0.14	-0.18	0.43^**^	0.24^*^	0.79^**^	-

Table 3 shows the mean difference of individuals with SUDs belonging to the nuclear and joint family systems at the risk of relapse, suicidal behavior, and ostracism, along with its dimension. The results indicate non-significant mean differences on all study variables (i.e., p > 0.05). This suggests that individuals with SUDs belonging to nuclear and joint family systems are equally vulnerable to the risk of relapse, and they also face similar types of ostracism. Additionally, Individuals with SUDs belonging to both types of family systems depict the same levels of suicidal behavior.

**Table 3:** The mean difference in study variables across family systems (N =100). M=mean, SD=standard deviation, LL=lower limit, UL=upper limit, CI=confidence interval, n=number of participants

Variables	Nuclear (n =62)		Joint (n = 38)		t (98)	p	95% CI	
	M	SD	M	SD			LL	UL
Risk of relapse	156.48	9.63	156.97	8.39	0.26	0.80	-3.26	4.24
Suicidal behavior	12.18	2.97	12.21	3.16	0.05	0.96	-1.21	1.28
Ostracism-ignored	9.16	2.74	9.53	3.05	0.62	0.54	-0.80	1.53
Ostracism-excluded	22.77	2.07	22.87	2.08	0.22	0.83	-0.75	0.94
Ostracism, total	31.82	3.56	32.16	4.14	0.43	0.67	-1.21	1.89

Table 4 shows the differences in all the study variables, including risk of relapse, suicidal behavior, and ostracism, along with its dimension across a family history of drug use. The "yes" indicates a number of people with an addiction with a family history of addiction. A total of 34 patients had someone in the family using drugs, whereas a more significant portion of the sample, i.e., 66 had no family history of drug usage. The results, however, showed no significant differences in the study variables across family history of drug usage. This suggests that individuals with SUDs with or without a family history of addiction are equally vulnerable to the risk of relapse, and they also face similar types of ostracism. Additionally, they also depict the same levels of suicidal behavior.

**Table 4:** The mean difference in study variables across the family history of drug use (N =100). M=mean, SD=standard deviation, LL=lower limit, UL =upper limit, CI= confidence interval, n=number of participant.

Variables	Yes (n =34)		No (n =66)		t (98)	p	95% CI	
	M	SD	M	SD			LL	UL
Risk of relapse	154.41	10.27	157.83	8.35	-1.79	0.08	-7.21	0.37
Suicidal behavior	11.94	3.02	12.32	3.04	-0.59	0.56	-1.65	0.89
Ostracism-ignored	9.41	3.26	9.24	2.64	0.28	0.78	-1.03	1.37
Ostracism-excluded	22.56	1.93	22.94	2.13	-0.87	0.38	-1.25	0.48
Ostracism, total	31.91	3.56	31.97	3.91	-0.07	0.94	-1.65	1.53

Table 5 shows the results of multiple hierarchical linear regression analysis. The analysis was conducted to predict the risk of relapse due to the ostracism faced by addicts while controlling for the effect of demographics. The results showed that none of the demographics significantly predicted the risk of relapse in the first step of hierarchical linear regression analysis. In the second step, both dimensions of ostracism were included in the regression analysis, and the result showed that both dimensions of ostracism significantly increased the risk of relapse (B ranging from 0.74 to 1.26) for ostracism-ignored and -excluded, respectively. The results, however, showed that ostracism-excluded is a stronger predictor of the risk of relapse as compared to ostracism-ignored. In the second step of the regression analysis, income also appeared to be a significantly negative predictor of the risk of relapse, suggesting that high-income people are at a lower risk of relapse, which may be because high-income people face less ostracism. The regression model explains a 22% risk of relapse, suggesting ostracism is one the major contributors to increasing the risk of relapse.

**Table 5:** Linear regression to predict the risk of relapse by ostracism-ignored and ostracism-excluded controlling for the effect of demographic (N =100). LL=lower limit, UL=upper limit, CI=confidence interval, B=Beta coefficient, ** p< 0.01 was considered of statistical significance.

Independent Variables	Model-1 B	Model-2		
		B	95% CI	
			LL	UL
Constant	157.03**	118.66**	87.73	149.60
Age	0.30	0.29	-0.28	0.86
Age of onset	-0.29	-0.14	-0.59	0.30
Family system	-0.05	0.41	-3.04	3.87
Monthly income	-0.08	-0.10*	-0.21	0.00
Ostracism-ignored	-	0.74*	0.10	1.38
Ostracism-excluded	-	1.26**	0.40	2.13
R^2^	0.05	0.22	-	-
ΔR^2^	-	0 .17	-	-
F	1.24	4.39**	-	-
ΔF	-	10.21**	-	-

Table 6 also shows the results of multiple hierarchical linear regression analysis. The analysis was conducted to predict suicidal behavior due to the ostracism faced by individuals with SUDs and their risk of relapse while controlling for the effect of demographics. The results showed that none of the demographics significantly predicted the risk of relapse in the first step of hierarchical linear regression analysis. In the second step, both dimensions of ostracism and the risk of relapse were included in the regression analysis, and the result showed that only ostracism-excluded (B = 0.21, p < 0.01) along with the risk of relapse (B = 0.11, p < 0.01) significantly increased suicidal behavior among substance users. Ostracism-ignored appeared not to affect suicidal behaviors (p > 0.05). The regression model explains a 19% variance in the suicidal behavior of substance users, suggesting ostracism-excluded and risk of relapse are important contributors to increasing suicidal behavior among substance users.

**Table 6:** Linear regression to predict suicidal behavior by risk of relapse, ostracism-ignored, and ostracism-excluded controlling for the effect of demographics (N =100). LL=lower limit, UL=upper limit, CI=confidence interval, B=Beta coefficient, * p < 0.05, ** p < 0.01 was considered of statistical significance.

Independent Variables	Model-1 B	Model-2		
		B	95% CI	
			LL	UL
Constant	8.88*	-13.74*	-27.15	-0.33
Age	0.12	0.09	-0.10	0.29
Age of onset	-0.01	0.03	-0.12	0.18
Family system	0.00	0.06	-1.12	1.23
Monthly income	-0.01	0.00	-0.04	0.03
Risk of relapse	-	0.11**	0.04	0.18
Ostracism-ignored	-	0.06	-0.16	0.28
Ostracism-excluded	-	0.21**	0.09	0.52
R^2^	0.02	0.19	-	-
ΔR^2^	-	0.17	-	-
F	0.37	2.99**	-	-
ΔF	-	6.40**	-	-

Table 7 shows the results of the mediation analysis. The analysis was conducted using process macro (version 3.1) in SPSS. To test mediation of the risk of relapse for the relationship between ostracism and suicidal behavior, the model no. 1 of the process macro was used. The model can accommodate up to 10 mediators simultaneously. However, only one predictor and outcome can be used in the mediation model. Both the dimensions of ostracism have the same direction of relationship with the predictor (i.e., risk of relapse) and the outcome (i.e., suicidal behavior). Hence, only the total score of the ostracism was used as a predictor variable. The results showed that risk ostracism significantly increased suicidal behaviors (B = 0.19, p < 0.05). The risk of relapse significantly positively mediated the relationship between ostracism and suicidal behavior. The analysis of direct and indirect effects showed that after incorporating the risk of relapse as a mediator in the analysis, there is no significant direct effect of ostracism on suicidal behavior. The effect is mediated through the risk of relapse (B indirect = 0.12, 95% CI = 0.04, 0.23). The results show that ostracism contributes to suicidal behavior by increasing the risk of relapse. The mediation model explained a total of 17% variance in suicidal behavior among individuals with substance use disorder.

**Table 7:** Linear regression to test the mediating role of risk of relapse for the relationship between ostracism and suicidal behavior (N =100). LL=lower limit, UL=upper limit, CI=confidence interval, B=Beta coefficient, * p < 0.05, ** p < 0.01 was considered of statistical significance.

Independent Variables	Model-1 B	Model-2		
		B	95% CI	
			LL	UL
Constant	2.86	-11.67	-24.16	0.83
Age	0.08	0.07	-0.12	0.26
Age of Onset	0.02	0.03	-0.12	0.19
Monthly Income	-0.01	-0.01	-0.04	0.04
Family History	0.34	-0.06	-1.28	1.16
Ostracism Total	0.19*	0.06	-0.11	0.24
Risk of Relapse	-	0.12**	0.05	0.19
R^2^	0.07	0 .17	-	-
ΔR^2^	-	0.10	-	-
F	1.40	3.10**	-	-
Indirect Effect	-	0.12	0.04	0.23
Direct Effect	-	0.06	-0.11	0.24
Total Effect	-	0.19	0.02	0.39

**Figure 1:**
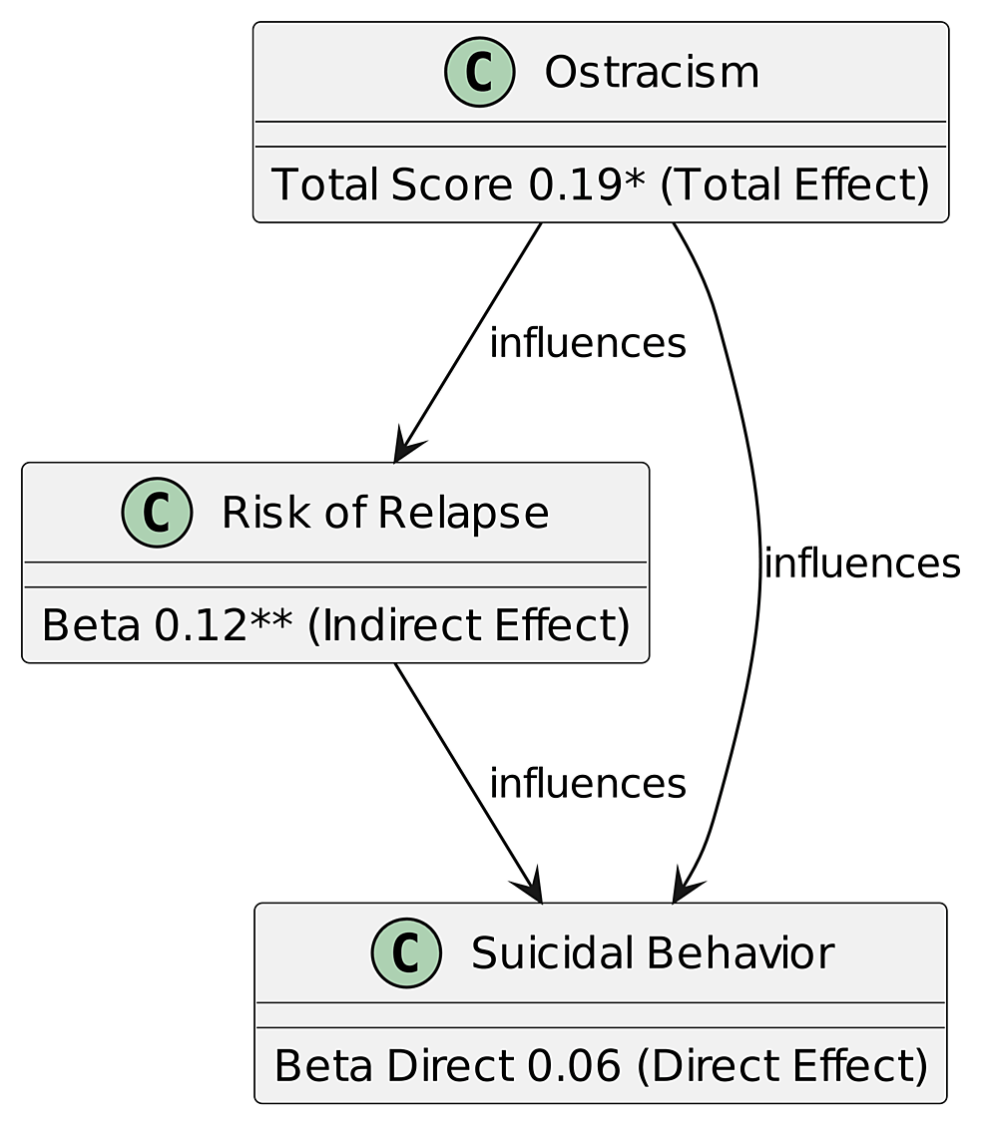
Mediation analysis of the role of risk of relapse in the relationship between ostracism and suicidal behavior. ** p < 0.01, *p<0.05 was considered of statistical significance.

Figure 1 illustrates the direct and indirect effects of ostracism on suicidal behavior, mediated by the risk of relapse. The total effect of ostracism on suicidal behavior is 0.19, while the indirect effect through risk of relapse is 0.12. The direct effect of ostracism on suicidal behavior, excluding the mediator, is 0.06.

## Discussion

Substance use is a global issue influenced by personal, environmental, and psychosocial factors, causing physical, psychological, and economic consequences [20]. Individuals often face social and community rejection, family rejection, and societal stigma, leading to increased addictive behavior. A study reveals that suicidal individuals often suffer from trauma from physical abuse and social exclusion, leading to factors like partner infidelity, hopelessness, social mocking, and ostracism affecting people with Substance Use Disorders (SUDs) [21]. Intervention plans target drug addiction relapse risk, focusing on family therapy and patient adherence improvement [22, 23]. Pakistan's sociocultural and psychological landscape significantly impacts individuals who experience SUDs, leading to ostracism, relapse risk, and suicidal behaviors due to stigma [24]. Research conducted by local populations emphasizes the need for culturally sensitive interventions [25].

The study evaluated the psychometric properties of three scales, revealing good internal consistency with alpha values ranging from 0.74 to 0.85. The study found high reliability in the OES and AWARE Questionnaire but low reliability in the SBQ-R, suggesting that the risk of relapse may play a mediating role between ostracism and suicidal behaviors. The study found no significant differences in relapse risk, suicidal behavior, or ostracism across family systems and drug use histories among substance users. Both nuclear and joint family systems were equally vulnerable to relapse and suicidal behavior. The majority of patients had a family history of drug use, but no significant differences were found across family history. Multiple hierarchical regression analysis was used to predict relapse risk in addicts due to ostracism; the results presented in tables 5-7 are supported by recent empirical investigations. A study by McGuire and Raleigh (1986) [26] shows that social rejection can lead to increased mood swings and fury, and long-term ostracism can result in attempted suicide, despair, and even mass killings [27].

The present study found that demographic variables did not significantly predict the risk of relapse in the first step of hierarchical linear regression analysis. However, when both dimensions of ostracism were included, both dimensions significantly increased the risk of relapse. Income was also found to be a negative predictor of relapse risk, suggesting that high-income individuals face less ostracism. The regression model explained 22% of the risk of relapse, confirming the first hypothesis.

To test the second hypothesis, multiple hierarchical linear regression analysis was conducted to predict suicidal behavior due to ostracism faced by addicts and their risk of relapse. There is a scarcity of literature exploring the relationship between ostracism and relapse risk, possibly due to its rarity in SUDs. However, empirical evidence suggests an increase in relapse risk in individuals with higher levels of ostracism. Relapse treatment focuses on critical issues and alters behavior to support progress made during treatment or self-change [28].

This study found that demographic factors did not significantly predict the risk of relapse in substance users. However, when ostracism (B = 0.21, p < 0.01) and risk of relapse (B = 0.11, p < 0.01) were included in the regression analysis, only ostracism-excluded significantly increased suicidal behavior among substance users. Ostracism-ignored did not affect suicidal behaviors (p > 0.05). The regression model explained a 19% variance in substance users' suicidal behavior, indicating that ostracism-excluded and risk of relapse are essential contributors. The mediation analysis, conducted using process macro in SPSS, showed that the risk of ostracism significantly increased suicidal behaviors. The risk of relapse significantly positively mediated the relationship between ostracism and suicidal behavior. These results confirm the second hypothesis of the study.

Research suggests that patients with Substance Use Disorder (SUD) may experience heightened impulsivity, leading to an increased risk of suicidal attempts. Clinicians should screen for relapse and design appropriate treatments to reduce this risk [29]. Suicidal tendencies may indicate elevated levels of impulsivity and sadness, which are significant predictors of recurrence [30]. Examining past suicidal behavior can identify individuals with severe psychopathology and a higher risk for relapse. Ostracism, when mediated through the risk of relapse, contributes to 17% of substance users' suicidal behavior. The low variances suggest that while ostracism and the risk of relapse are significant predictors, there are likely other contributing factors influencing suicidal behavior among substance users that were not captured in our models. Future research should aim to identify additional variables that could explain a greater proportion of the variance in suicidal behavior. Mental health professionals should use this information to inform early intervention strategies, including cognitive behavioral therapy [31], support groups [32], and mindfulness-based practices [33] to help individuals manage emotions and stress.

The study's cross-sectional design limits causality and a longitudinal design is recommended for better results. The sample included 100 substance users from Karachi, and there was a need for a national-level sample. The study focused on male substance users, neglecting females. It also did not test the hypothesis across different drug types or moderators, suggesting a need for further research. Future studies should consider moderators for a more comprehensive understanding.

## Conclusions

It is concluded that ostracism in all forms is a risk factor that increases the risk of relapse among people with SUDs. To improve the stable recovery of substance users, community orientation plans should be initiated to promote awareness against the stigmatization of SUDs. Additionally, an increased risk of relapse is associated with an increase in suicidal behaviors, and hence, the intervention plans need to pay particular focus to effective relapse prevention strategies. Social rejection, psychological stress, social pressure, and socioeconomic status, such as drug accessibility and availability, peer group influences, and a lack of assertiveness, were risk factors for the prevalence of risk of relapse and suicidal behaviors. Drug use treatment should emphasize long-term follow-up rather than just detoxification to prevent relapse. To enhance patient outcomes, post-discharge relapse prevention measures were recommended. Additionally, it is crucial for the national Ministry of Health to evaluate the effectiveness of existing relapse prevention strategies and their implementation. Developing a standardized relapse prevention program could significantly reduce the high incidence of recurrence. Also, several sensitization campaigns should be conducted to increase public awareness of the burden of substance use, the consequences of relapse after treatment, and their effects not only on individual health but also on the health of the community.

## Appendices

Appendix A

The Ostracism Experience Scale (OES), as presented in Table 18, is an 11-item five-point Likert scale with an alpha reliability of 0.97, providing insights into social exclusion [17]. 

**Table 8:** Ostracism Experiences Scale

S.No	Items	Hardly ever	………………………			Almost always
		1	2	3	4	5
1.	Others treat me as if I am invisible					
2.	Others look through me as if I do not exist					
3.	Others have ignored my greetings when we are walking by					
4.	Others ignore me during conversation					
5.	Others ignore me					
6.	Others “hang out” with me at my home					
7.	Others invite me to join their club, organization, or association					
8.	Others include me in their plans for the holidays					
9.	Others make an effort to get my attention					
10.	Others invite me to go out to eat with them					
11.	Others invite me to join them for weekend activities, hobbies, or events					

Appendix B

The Advance Warning of Relapse Questionnaire 3.0 (AWARE), as depicted in Table 9, has been refined to include 28 items on a seven-point Likert scale, effectively predicting relapse (r = 0.42, p < 0.001) following outpatient alcohol treatment [18]. 

**Table 9:** The AWARE Questionnaire (Advance WArning of RElapse). Please circle one number for every statement.

	Never	Rarely	Some times	Fairly often	Often	Almost always	Always
1. I feel nervous or unsure of my ability to stay sober.	1	2	3	4	5	6	7
2. I have many problems in my life.	1	2	3	4	5	6	7
3. I tend to overreact or act impulsively.	1	2	3	4	5	6	7
4. I keep to myself and feel lonely.	1	2	3	4	5	6	7
5. I get too focused on one area of my life.	1	2	3	4	5	6	7
6. I feel blue, down, listless, or depressed.	1	2	3	4	5	6	7
7. I engage in wishful thinking.	1	2	3	4	5	6	7
8. The plans that I make succeed.	1	2	3	4	5	6	7
9. I have trouble concentrating and prefer to dream about how things could be.	1	2	3	4	5	6	7
10. Things don’t work out well for me.	1	2	3	4	5	6	7
11. I feel confused.	1	2	3	4	5	6	7
12. I get irritated or annoyed with my friends.	1	2	3	4	5	6	7
13. I feel angry or frustrated.	1	2	3	4	5	6	7
14. I have good eating habits.	1	2	3	4	5	6	7
15. I feel trapped and stuck, like there is no way out.	1	2	3	4	5	6	7
16. I have trouble sleeping.	1	2	3	4	5	6	7
17. I have long periods of serious depression.	1	2	3	4	5	6	7
18. I don’t really care what happens.	1	2	3	4	5	6	7
19. I feel like things are so bad that I might as well drink.	1	2	3	4	5	6	7
20. I am able to think clearly.	1	2	3	4	5	6	7
21. I feel sorry for myself.	1	2	3	4	5	6	7
22. I think about drinking.	1	2	3	4	5	6	7
23. I lie to other people.	1	2	3	4	5	6	7
24. I feel hopeful and confident.	1	2	3	4	5	6	7
25. I feel angry at the world in general.	1	2	3	4	5	6	7
26. I am doing things to stay sober.	1	2	3	4	5	6	7
27. I am afraid that I am losing my mind.	1	2	3	4	5	6	7
28. I am drinking out of control.	1	2	3	4	5	6	7

Appendix C

The Suicide Behaviors Questionnaire-Revised (SBQ-R), as shown in Table 10, a four-item scale with high internal consistency (α = 0.97), offered a concise yet robust self-report measure for past suicidal behaviors [19]. 

**Table 10:** SBQ-R Suicide Behaviors Questionnaire-Revised.

Serial No	Item	Response
1	Have you ever thought about or attempted to kill yourself? (check one only)	-
	1 Never	
	2 It was just a brief passing thought	
	3a. I have had a plan at least once to kill myself but did not try to do it	
	3b. I have had a plan at least once to kill myself and really wanted to die	
	4a. I have attempted to kill myself, but did not want to die	
	4b. I have attempted to kill myself, and really hoped to die	
2	How often have you thought about killing yourself in the past year? (check one only)	-
	1. Never	
	2. Rarely (1 time)	
	3. Sometimes (2 times)	
	4. Often (3-4 times)	
	5. Very Often (5 or more times)	
3	Have you ever told someone that you were going to commit suicide, or that you might do it? (check one only)	-
	1. No	
	2a. Yes, at one time, but did not really want to die 2b. Yes, at one time, and really wanted to die	
	3a. Yes, more than once, but did not want to do it	
	3b. Yes, more than once, and really wanted to do it	
4	How likely is it that you will attempt suicide someday? (check one only)	-
	0. Never	
	1. No chance at all	
	2. Rather unlikely	
	3. Unlikely	
	4. Likely	
	5. Rather likely	
	6. Very likely	
